# Subacute Intranasal Administration of Tissue Plasminogen Activator Promotes Neuroplasticity and Improves Functional Recovery following Traumatic Brain Injury in Rats

**DOI:** 10.1371/journal.pone.0106238

**Published:** 2014-09-03

**Authors:** Yuling Meng, Michael Chopp, Yanlu Zhang, Zhongwu Liu, Aaron An, Asim Mahmood, Ye Xiong

**Affiliations:** 1 Department of Neurosurgery, Henry Ford Hospital, Detroit, Michigan, United States of America; 2 Department of Neurology, Henry Ford Hospital, Detroit, Michigan, United States of America; 3 Department of Physics, Oakland University, Rochester, Michigan, United States of America; University of Rouen, France

## Abstract

Traumatic brain injury (TBI) is a major cause of death and long-term disability worldwide. To date, there are no effective pharmacological treatments for TBI. Recombinant human tissue plasminogen activator (tPA) is the effective drug for the treatment of acute ischemic stroke. In addition to its thrombolytic effect, tPA is also involved in neuroplasticity in the central nervous system. However, tPA has potential adverse side effects when administered intravenously including brain edema and hemorrhage. Here we report that tPA, administered by intranasal delivery during the subacute phase after TBI, provides therapeutic benefit. Animals with TBI were treated intranasally with saline or tPA initiated 7 days after TBI. Compared with saline treatment, subacute intranasal tPA treatment significantly 1) improved cognitive (Morris water maze test) and sensorimotor (footfault and modified neurological severity score) functional recovery in rats after TBI, 2) reduced the cortical stimulation threshold evoking ipsilateral forelimb movement, 3) enhanced neurogenesis in the dentate gyrus and axonal sprouting of the corticospinal tract originating from the contralesional cortex into the denervated side of the cervical gray matter, and 4) increased the level of mature brain-derived neurotrophic factor. Our data suggest that subacute intranasal tPA treatment improves functional recovery and promotes brain neurogenesis and spinal cord axonal sprouting after TBI, which may be mediated, at least in part, by tPA/plasmin-dependent maturation of brain-derived neurotrophic factor.

## Introduction

Traumatic brain injury (TBI) remains a leading cause of mortality and morbidity worldwide [1], [2]. To date, there is no effective pharmacological therapy available for TBI. For decades, significant efforts have been devoted to the development of neuroprotective agents in an attempt to prevent neural cell death or salvage damaged neurons in the injured brain; however, all these efforts have failed to demonstrate efficacy in clinical trials of TBI [1], [3]. Until recently, it was believed that once the brain was damaged, there was little, if any, capability for regeneration of axons and formation of new synapses. However, it has been discovered that the central nervous system (CNS) is indeed capable of significant (though limited) plasticity and regeneration that may contribute to spontaneous functional recovery and can be pharmacologically or otherwise enhanced [4]–[8].

Recombinant human tissue plasminogen activator (tPA) is the only U.S. Food and Drug Administration approved drug for the treatment of acute ischemic stroke [9]. In addition to its well established thrombolytic effect, tPA also participates in synaptic plasticity, dendritic remodeling and axonal outgrowth in the developing and injured CNS [10]. Our previous studies have demonstrated that upregulated endogenous tPA mediates bone marrow stromal cell-induced functional recovery in animal models of stroke [11] and TBI [12]. tPA is able to convert the pro-brain-derived neurotrophic factor (proBDNF) to the mature BDNF by activating the extracellular protease plasmin, and that such conversion is critical for brain neuroplasticity and function [13]–[17]. However, tPA has potential adverse side effects when administered intravenously including brain edema and hemorrhagic transformation in rats after stroke [9] and increased brain lesion and hemorrhage in rats after TBI [18]. Intranasal delivery of agents has been demonstrated to directly target the brain and spinal cord [19]. Although the exact mechanisms of this intranasal delivery are not yet fully understood, extensive evidence demonstrates that olfactory nerve pathways, trigeminal nerve pathways, vascular pathways and lymphatic pathways are involved [19]. Our previous study demonstrates that tPA administrated intranasally during the subacute phase of experimental stroke in rats provides beneficial effects on stroke recovery by promoting neuroplasticity [20]. However, there is no study of subacute intranasal tPA as a potential treatment of TBI. Whether and how intranasal tPA administration after TBI regulates BDNF is unknown.

In the present study, we investigated the therapeutic effect of tPA administered intranasally on cognitive and sensorimotor functional recovery in rats during the subacute phase of experimental TBI. We performed intracortical microstimulation (ICMS) that evoked right or left forelimb movement to validate the establishment of functional neuronal connections from the right intact cortex to bilateral forelimbs 5 weeks after TBI. We examined the effects of tPA treatment on neurogenesis in the dentate gyrus (DG) and axonal sprouting of the corticospinal tract (CST) originating from the intact cortex into the denervated side of the spinal cord after TBI to investigate the neuronal substrate of the functional recovery. To elucidate the mechanisms that underlie the beneficial effects of tPA, we investigated expression of proBDNF and BDNF in the injured brain and denervated cervical spinal cord in TBI rats treated with tPA. Here we report that subacute intranasal tPA treatment improves functional recovery and promotes brain neurogenesis and spinal cord axonal sprouting after TBI in rats, which is likely associated with tPA/plasmin-dependent maturation of BDNF.

## Materials and Methods

### Ethics Statement

All experimental procedures were carried out in accordance with the NIH Guide for the Care and Use of Laboratory Animals. The study protocol was approved by the Institutional Animal Care and Use Committee of Henry Ford Hospital.

### TBI Model

A controlled cortical impact (CCI) model of TBI in the rat was utilized for the present study [21]. Young adult male Wistar rats (323±13 g) were anesthetized with chloral hydrate (350 mg/kg body weight), intraperitoneally. Rectal temperature was maintained at 37°C using a feedback-regulated water-heating pad. Rats were placed in a stereotactic frame. Two 10-mm-diameter craniotomies were performed adjacent to the central suture, midway between lambda and bregma. The second craniotomy allowed for lateral movement of cortical tissue. The dura mater was kept intact over the cortex. To verify the neuroanatomical substrate of sensorimotor functional recovery after TBI and tPA treatment, we injected an anterograde neuronal tracer biotinylated dextran amine (BDA) into the contralesional right sensorimotor cortex [5], [22]. In brief, 10% solution of BDA (10,000 MW; Molecular Probes, Eugene, OR) in phosphate-buffered saline (PBS) was injected through a finely drawn glass capillary into 4 points in the right cortex (100 nl per injection; stereotaxic coordinates: 1 and 2 mm posterior to the bregma, 3 and 4 mm lateral to the midline, at a depth of 1.5 to 1.7 mm from the cortical surface) to anterogradely label the CST originating from this area. The micropipette remained in place for 4 min after completion of the injection. Immediately after BDA injection, cortical injury was delivered by impacting the left cortex (ipsilateral cortex) with a pneumatic piston containing a 6-mm-diameter tip at a rate of 4 m/s and 2.5 mm of compression. Velocity was measured with a linear velocity displacement transducer [5].

### Experimental Groups and Treatment

Young adult male Wistar rats subjected to TBI were randomly divided into 2 groups: TBI+Saline group (*n* = 8) and TBI+tPA group (*n* = 8). TBI was induced by CCI over the left parietal cortex. Sham+tPA rats (*n* = 8) underwent surgery without injury but received the same dose of intranasal tPA as did the TBI+tPA rats. tPA at a dose of 600 µg/rat (Genentech, San Francisco, CA) was administered intranasally at Days 7 and 14 post TBI or sham-operation. Although our pilot study did not show any significant effect of intranasal delivery of tPA (600 µg/rat) on functional outcomes in sham-operated rats, all the sham animals received intranasal tPA (600 µg/rat) post sham-operation for functional tests, Western blot, enzymatic and immunostaining analyses. The starting dose of tPA was selected based on our previous stroke study in rats [20]. Intranasal delivery does not involve delivery to the bloodstream throughout the body where the body weight is a significant factor, but rather targets the brain and spinal cord [23]. Thus throughout the proposal, we used micrograms (i.e., µg) to express drug dosage administered to the rats. Animals in the saline-treated group received an equal volume of saline. Briefly, under isoflurane anesthesia, the animals were placed in a supine position with rolled 2×2 inch gauze under the neck to maintain a horizontal head position [20]. Ten 6-µl drops for a total volume of 60 µl of saline or tPA solution in saline were placed alternately onto each nostril with a 3-min interval between drops and naturally sniffed in by the rat. The animals were kept in supine position for an additional 10 min. For labeling proliferating cells, 5-bromo-2′-deoxyuridine (BrdU, 100 mg/kg; Sigma, St. Louis, MO, USA) was injected intraperitoneally into rats daily for 10 days, starting 1 day after TBI. All rats were sacrificed at 35 days after TBI.

To investigate the molecular mechanisms by which tPA promotes neuroplasticity and functional recovery, we examined expression level of proBDNF and BDNF proteins in the injured brain cortex and denervated cervical spinal cord in rats after TBI treated with tPA (*n* = 8). The animals were treated intranasally with saline or one dose of 600 µg tPA administered at day 7 after TBI or sham-operation and sacrificed 24 hr later after tPA treatment for immunostaining and Western blot analysis of proBDNF and BDNF expression. To determine the efficiency of intranasal delivery and avoid the interference of endogenous tPA with the assay, we delivered 300 µg recombinant human tPA (ten 3-µl drops) intranasally into tPA knockout mice (Jackson Laboratory, Bar Harbor, ME). The mice were euthanized at 30 min or 120 min (n = 3/group) after the start of intranasal treatment, and their brains were then frozen in liquid nitrogen and stored at −80°C until use. Brain tissue samples were extracted with 50 mM phosphate-buffered saline (pH 7.4) containing 1% Triton X-100 and centrifuged at 14,000×g for 20 min. The supernatant was collected, and the protein concentration was measured bicinchoninic acid (BCA) protein assay (Pierce, Rockford, IL). The tPA content in the brain was quantitated with equal amounts of lysates from tissue samples using a human tPA total antigen enzyme-linked immunosorbent assay (ELISA) kit (Molecular Innovations, Novi, MI).

### Morris Water Maze (MWM) Test

All functional tests were performed by investigators blinded to the treatment status. To detect spatial learning impairments, a recent version of the MWM test was used [24]. The procedure was modified from previous versions [25] and has been found to be useful for spatial memory assessment in rodents with brain injury [24]. Animals were tested during the last 5 days (that is, 31–35 days after TBI) before sacrifice. Data collection was automated using the HVS Image 2020 Plus Tracking System (US HVS Image), as described previously [26]. The advantage of this version of the water maze is that each trial takes on the key characteristics of a probe trial because the platform is not in a fixed location within the target quadrant. In the traditional version of the MWM test, the position of the hidden platform is always fixed and is relatively easy for rodents to locate. With the modified MWM test we used in the present study, the platform is relocated randomly within the target quadrant (that is, Northeast) with each training trial. The rodents must spend more time searching within the target quadrant; therefore each trial effectively acts as a probe trial. The advantage of this protocol is that rodents should find the platform purely and extensively by reference to extra-maze spatial cues, which improves the accuracy of spatial performance in the MWM [24].

### Foot Fault Test

To evaluate sensorimotor function, the foot fault test was carried out before TBI and at 1, 7, 14, 21, 28 and 35 days after TBI. The rats were allowed to walk on a grid. With each weight-bearing step, a paw might fall or slip between the wires and, if this occurred, it was recorded as a foot fault [27]. A total of 50 steps were recorded for right forelimbs.

### Modified Neurological Severity Score (mNSS) Test

Neurological functional measurement was performed using the mNSS test [28]. The test was carried out on all rats preinjury and at 1, 7, 14, 21, 28 and 35 days after TBI. The mNSS is a composite of the motor (muscle status, abnormal movement), sensory (visual, tactile, and proprioceptive), and reflex tests and has been used in previous studies [29]. Neurological function was graded on a scale of 0 to 18 (normal score 0; maximal deficit score 18). In the severity scores of injury one point is awarded for each abnormal behavior or for lack of a tested reflex, thus, the higher the score, the more severe the injury.

### Electrophysiology

To validate the establishment of functional neuronal connections from the right intact cortex to bilateral forelimbs, intracortical microstimulation (ICMS) and electromyograms (EMG) were performed 5 weeks after TBI [30]. Rats were anesthetized with ketamine hydrochloride (100 mg/kg, intraperitoneally) and xylazine (5 mg/kg, intraperitoneally), and ketamine (20 mg/kg, intraperitoneally) as supplementary injections when needed. The animal rectal temperature was controlled at 37−C throughout the experiment. The animal was restricted in a Kopf stereotaxic apparatus and the craniotomy was performed over the right frontal motor cortex. The exposed cerebral cortex surface was covered with warm silicone oil. A pargylinecoated tungsten stimulating microelectrode (2.0 Megaohms, type TM33A20; WPI, Inc., Sarasota, FL) was descended into the forelimb motor cortex to a depth of 1.5 to 1.7 mm from the cortical surface to evoke movement at the lowest threshold at 4 points (stereotaxic coordinates: 1 and 2 mm rostral to the bregma, 2.5 and 3.5 mm lateral to the midline). The electrical stimulus consisted of 20 monophasic cathodal train pulses (60-ms duration at 333 Hz, 0.5-ms pulse duration) of a maximum of 100 µA. For every stimulating point, the lowest threshold value of ICMS that evoked right or left forelimb movement was measured. If no movements were evoked with 100 µA, the current threshold of this point was recorded as 100 µA without additional stimulation at a higher level to avoid cerebral tissue damage. If cortical vessels were encountered at the intended incision site, the site was moved immediately rostral or caudal to avoid the cortical vessel. The average threshold values evoking right or left forelimb movement were calculated from data of 4 stimulation points in each individual animal.

### Tissue Preparation

Rats were anesthetized at 8 or 35 days post TBI with chloral hydrate administered intraperitoneally and perfused transcardially with saline solution, followed by 4% paraformaldehyde in 0.1 M PBS, pH 7.4. Rat brains and cervical spinal cords were removed and immersed in 4% paraformaldehyde for 4 days. Using a rat brain matrix (Activational Systems, Inc., Warren, MI), each forebrain was cut into 2-mm-thick coronal blocks for a total of 7 blocks from bregma 5.2 mm to bregma −8.8 mm per animal [31]. For lesion volume measurement, one 6-*µ*m-thick section from each of 7 coronal blocks was traced by a microcomputer imaging device (MCID) (Imaging Research, St. Catharine's, Ontario, Canada), as described previously [32]. The volumes of the ipsilateral and contralateral cortices were computed by integrating the area of each cortex measured at each coronal level and the distance between two sections. The cortical lesion volume was expressed as a percentage calculated by [(contralateral cortical volume – ipsilateral cortical volume)/(contralateral cortical volume) ×100% [33]–[35]. This method has been widely used to measure lesion volume after TBI [34], [36]–[40] and after stroke [33], [35], [41], [42].

To visualize the BDA-labeled CST, the cervical spinal cord segments were processed for vibratome traverse sections (100 µm). Sections were incubated with 0.5% H_2_O_2_ for 20 min followed with avidin-biotin-peroxidase complex (Vector Laboratories Inc., Burlingame, CA) at 4°C for 48 h, and BDA-labeled axons were visualized with 3,3′-diaminobenzidine-nickel (Vector) for light microscopy examination [30]. For measuring axons crossing the midline of cervical spinal cord, the central canal and dorsal median fissure were used as landmarks with the number of labeled axons projecting into the denervated TBI-impaired side of the ventral gray matter. For each animal, the CST remodeling was estimated by the total BDA-labeled axonal length on 40 consecutive cervical cord sections (C4–7), as previously described [30].

### Immunohistochemistry

To examine the effect of tPA on neuroblasts, proBDNF and BDNF, immunostaining was performed in five brain coronal sections and cervical spinal cord transverse sections. Briefly, 6-µm paraffin-embedded sections were deparaffinized and rehydrated. Antigen retrieval was performed by boiling sections in 10-mM citrate buffer (pH 6.0) for 10 min. After washing with PBS, sections were incubated with 0.3% H_2_O_2_ in PBS for 10 min, blocked with 1% BSA containing 0.3% Triton-X 100 for 1 h at room temperature, and incubated with mouse anti-DCX (1∶200; Santa Cruz Biotechnology, Santa Cruz, CA) or rabbit anti-proBDNF (1∶200; AbCam, Cambridge, MA) or rabbit anti-BDNF (1∶200; Santa Cruz Biotechnology, CA) at 4°C overnight. For negative controls, primary antibodies were omitted. After washing, sections were incubated with biotinylated anti-mouse or anti-rabbit antibodies (1∶200; Vector Laboratories, Inc., Burlingame, CA) for 30 min at room temperature. After an additional washing, sections were incubated with an avidin-biotin-peroxidase system (ABC kit, Vector Laboratories, Inc.), visualized with diaminobenzidine (Sigma, St. Louis, MO), and counterstained with hematoxylin.

### Immunofluorescent Staining

Newly generated neurons in the DG were identified by double labeling for BrdU and NeuN after TBI [43]. Briefly, after being deparaffinized and rehydrated, tissue sections were boiled in 10 mM citric acid buffer (pH 6) for 10 min. After washing with PBS, sections were incubated in 2.4 N HCl at 37°C for 20 min. Sections were incubated with 1% BSA containing 0.3% Triton-X-100 in PBS. Sections were then incubated with mouse anti-NeuN antibody (1∶200; Chemicon, Temecula, CA) at 4°C overnight. For negative controls, primary antibodies were omitted. FITC-conjugated anti-mouse antibody (1∶400; Jackson ImmunoResearch, West Grove, PA) was added to sections at room temperature for 2 h. Sections were then incubated with rat anti-BrdU antibody (1∶200; Dako, Glostrup, Denmark) at 4°C overnight. Sections were then incubated with Cy3-conjugated anti-rat antibody (1∶400; Jackson ImmunoResearch, West Grove, PA) at room temperature for 2 h. Each of the steps was followed by three 5-min rinses in PBS. Tissue sections were mounted with Vectashield mounting medium (Vector laboratories, Burlingame, CA).

### Cell Counting and Quantitation

Although we did not use unbiased stereology to count cells in the present study, previous studies from us and other investigators have shown that the method used provides a meaningful comparison of differences in cell counting among groups after TBI and treatment [39], [44]–[51] and stroke [52]. Cell counts were performed by observers blinded to the individual treatment status of the animals. For quantitative measurements of immunostaining positive cells in brain, 5 coronal brain slides from each rat were employed, with each slide containing 5 fields of view from the lesion boundary zone from the epicenter of the injury cavity (bregma −3.3 mm), 3 fields of view from the ipsilateral CA3 and 9 fields of view from the ipsilateral DG in the same section. For quantitative measurements of immunostaining positive cells in cervical spinal cord (C4–7), 10 transverse sections from each rat were used. For analysis of proBDNF^+^ and BDNF^+^ cells, we focused on the ventral horn of the denervated cervical spinal cord and on the injured boundary zone of ipsilateral cortex. For analysis of neurogenesis, we focused on the ipsilateral DG and its subregions, including the subgranular zone, granular cell layer, and the molecular layer. The number of BrdU^+^ cells (red stained) and NeuN/BrdU-colabeled cells (yellow after merge) were counted in the DG. The percentage of NeuN/BrdU-colabeled cells over the total number of BrdU^+^ cells in the DG was estimated and used as a parameter to evaluate neurogenesis [53]. The fields of interest were digitized under the light microscope (Nikon, Eclipse 80i, Melville, NY) at a magnification of either 200 or 400 using CoolSNAP color camera (Photometrics, Tucson, AZ) interfaced with MetaMorph image analysis system (Molecular Devices, Downingtown, PA). The immunopositive cells were calculated and divided by the measured areas, and presented as numbers per square mm. All the counting was performed on a computer monitor to improve visualization and in one focal plane to avoid oversampling [54].

### Western Blot Analyses

Animals were sacrificed on Day 8 after TBI (24 hr later after tPA treatment). The ipsilateral cortical tissues from lesion boundary zone and denervated cervical spinal cord (right side of C1–7) were dissected, frozen in liquid nitrogen and stored at −80°C. Brain and spinal cord tissues were thawed and lysed in lysis buffer containing 20 mM Tris pH 7.6, 100 mM NaCl, 1% Nonidet P-40, 0.1% SDS, 1% deoxycholic acid, 10% glycerol, 1 mM EDTA, 1 mM NaVO_3_, and 50 mM NaF (Calbiochem, San Diego, CA). After sonication, soluble protein was obtained by centrifugation at 13,000×g for 15 min at 4°C. Total protein concentrations were determined with bicinchoninic acid (BCA) protein assay (Pierce, Rockford, IL). Equal amounts of lysate were subjected to SDS-polyacrylamide electrophoresis on Novex tris-glycine pre-cast gels (Life Technologies, Grand Island, NY) and separated proteins were then electrotransferred to polyvinylidene fluoride (PVDF) membranes (Millipore, Bedford, MA). After exposure to various antibodies, specific proteins were visualized using SuperSignal West Pico chemiluminescence substrate system (Pierce Rockford, IL). Antibodies used for Western blot included anti-proBDNF (1∶1000; AbCam, Cambridge, MA), anti-BDNF (1∶1000; Santa Cruz Biotechnology, Santa Cruz, CA), anti-tPA (1∶2000; H-90: sc-15364, Santa Cruz Biotechnology, Santa Cruz, CA), and anti-Actin (1∶5000, Santa Cruz Biotechnology, Santa Cruz, CA). The band intensity was analyzed using Scion image software (Scion, Frederick, MD) [55].

### ELISA Assay of Human tPA

To determine the efficiency of intranasal delivery, tPA knockout mice weighing 22–25 g (n = 3/group, Jackson Laboratory, Bar Harbor, ME) were treated with 300 µg recombinant human tPA (ten 3-µl drops) intranasally. The mice were euthanized at 30 min or 120 min after the start of intranasal and brain extracts prepared for human tPA total antigen assay. The human tPA content in the brain of tPA knockout mice was quantitated using a Human tPA Total Antigen ELISA Assay kit (Molecular Innovations, Novi, MI) according to the manufacturer's recommendations. Free and complexed human tPA were detected by the assay. This kit does not cross react with mouse or rat tPA. Brain extract samples were performed with the 96-well strip format ELISA kit. Human tPA bound to the capture antibody coated on the microtiter plate. After appropriate washing steps, anti-human tPA primary antibody bound to the captured protein. Excess primary antibody was washed away and bound antibody was reacted with the secondary antibody conjugated to horseradish peroxidase. Following an additional washing step, tetramethylbenzidine chromogenic substrate was used for color development at 450 nm. Color development was proportional to the concentration of human tPA in the brain samples. A standard calibration curve was prepared in blocking buffer using dilutions of purified human tPA and was measured along with the test samples. The human tPA content was represented as ng/g wet brain tissue.

### Amidolytic Assay for Plasmin Activity

We performed colorimetric assays of plasmin in the lysates of brain tissues using D-Val-Leu-Lysp-Nitroanilide Dihydrochloride (S-2251) (Sigma-Aldrich, Saint Louis, MO) as a specific substrate for plasmin [56]. At the time of assay, the frozen brain tissues were thawed on ice, minced in sample buffer containing 50 mM Tris (pH 7.7), 0.2% (v/v) Triton X-100, 50 mM NaCl and 3 mM EDTA. Brain homogenates were prepared as described above for Western blot. To 5 µl of brain total protein extracts (23–50 µg protein), we added 55 µl of assay medium including 1.2 mM S-2251 according to the manufacture's instructions. The samples were incubated at 37°C in flat-bottomed 96-well microtiter plates and monitored the release of para-nitroaniline (pNA) in each well at 405 nm with a micro-ELISA auto reader (Fusion TM AFUSO model, Perkins Elmer Inc, Shelton, CT, USA). Each sample was measured in duplicate. Plasmin enzymatic activity was calculated based on the change of optical density over time, then normalized by protein content, and data were expressed as percentage of increase as compared to sham values.

### Indirect Amidolytic Assay for tPA Activity

We performed indirect measurement of tPA activity in the lysates of brain tissues, based on an amidolytic assay with addition of plasminogen to the assay system where tPA cleaves plasminogen to plasmin, by assessing plasmin activity on S-2251, as described previously based on an amidolytic assay that detects the activation of plasminogen to plasmin that cleaves S-2251 to form pNA, as described previously [56]–[58]. The change in absorbance of the pNA in the reaction solution at 405 nm is directly proportional to the enzymatic activity of plasmin generated by tPA through cleavage of plasminogen. Measurements were performed on brain total protein extracts prepared as above described for plasmin activity assay. To 5 µl of brain extracts, we added 55 µl of assay medium including 1.2 mM S-2251 and plasminogen (4 µg/ml). The samples were incubated at 37°C in flat-bottomed microtiter plates and monitored the release of pNA in each well at 405 nm with a micro-ELISA auto reader (Fusion TM AFUSO model; Perkins Elmer Inc, Shelton, CT, USA). Each sample was measured in duplicate. tPA enzymatic activity was calculated based on the change of optical density over time, then normalized by protein content and data were expressed as percentage of increase as compared to sham values.

### Direct Zymography Assay for tPA Activity

Using a human recombinant tPA (h-r-tPA) as a positive control to distinguish tPA from uPA, we measured tPA activity by direct zymography, as described previously by us [11], [55], [59] and others [60], [61]. Briefly, 25 µg protein samples were mixed with the sample loading buffer without β-mercaptoethanol, and heating was omitted. The mixture of the lower gel (10% acrylamide) contained casein (1 mg/ml, Sigma) and plasminogen (13 µg/ml, American Diagnostica, Greenwich, CT) as substrates for plasmin and tPA, respectively. The gel was then washed for 30 min with 2.5% Triton X-100 to remove SDS and further washed for 10 min with 0.1 M Tris buffer, pH 8. The new Tris buffer was replaced and the gel was incubated for 4 hrs at 37°C to allow caseinolysis occur. On the darkly stained casein background, tPA activity was visualized as light bands resulting from casein degradation. To verify tPA and distinguish from uPA, human recombinant tPA (15 ng, Genentech) was used a positive control. To verify loading variations, duplicate samples were used in gel electrophoresis. After electrophoresis, the gel was stained with Coomassie Blue R-250 and destained with 40% methanol as well as 10% acetic acid.

### Statistical Analyses

All data are presented as the means + standard deviation. Data were analyzed by analysis of variance (ANOVA) for repeated measurements of functional tests (spatial performance and sensorimotor function). For lesion volume, cell counting, axonal sprouting, Western blot data, plasmin and tPA activities, a one-way ANOVA followed by post hoc Student-Newman-Keuls tests was used to compare the difference between the tPA-treated, saline-treated and sham groups. Pearson's correlation coefficients were calculated between functional recovery and anatomic reorganization. Statistical significance was set at *p*<0.05.

## Results

### tPA Protein Level and Activity in Brain Extracts after Intranasal Administration of tPA

**Figure 1:**
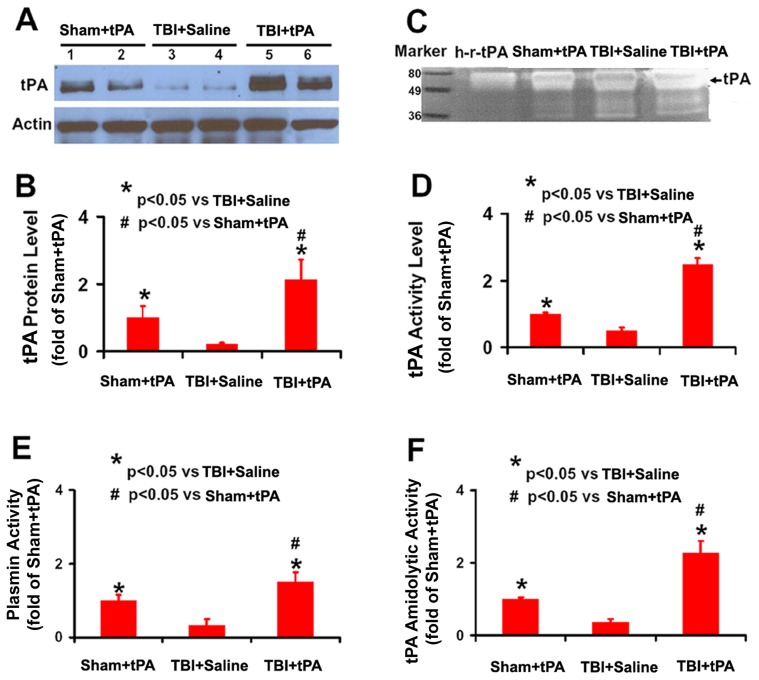
tPA protein level and activity as well as plasmin activity in rat brain. Western blot analysis of tPA protein levels in the rat brain 24 hr after intranasal administration of tPA (A). Bar graph (B) shows the tPA protein level. Of note, Sham+tPA rats received the same amount of intranasal tPA administration as TBI+tPA rats did. Representative zymographic assay (C) shows an increase in tPA activity in the Sham+tPA rats and TBI+tPA rats compared to TBI+Saline rats. h-r-tPA: human recombinant tPA (15 ng, Genentech). Bar graph (D) shows the tPA activity. Bar graph (E) shows amidolytic activity of plasmin assayed with D-Val-Leu-Lys-p-Nitroanilide Dihydrochloride (S-2251) as its specific substrate. Bar graph (F) shows tPA amidolytic activity with S-2251 as the substrate in the presence of added plasminogen compared to Sham. *p<0.05 vs TBI+Saline. ^#^ *p*<0.05 vs Sham+tPA. Data represent mean ± SD. n = 4 (rats/group, in graphs D, E, and F).

**Figure 2:**
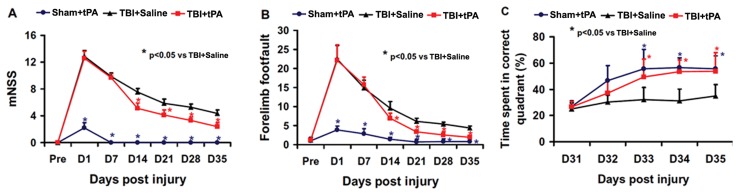
tPA effect on functional outcome. tPA significantly lowered the mNSS scores (A) and reduced frequency of foot faults (B) from Day 14 to 35 after TBI compared to the saline group. tPA treatment significantly improved spatial learning performance from Day 33 to 35 after TBI compared with the saline group (C). *p<0.05 vs TBI+Saline. Data represent mean ± SD. n = 8 (rats/group).

In this study, to avoid potential interference of endogenous tPA with the assay, we first administered human recombinant tPA intranasally into tPA knockout mice. The concentrations of total human tPA were 307±10 ng/g and 228±67 ng/g in brain tissue at 30 and 120 min, respectively after tPA delivered intranasally, indicating that intranasal delivery is an efficacious method to deliver tPA into the brain and a considerable amount of tPA remains in the brain at least up to 2 h after intranasal administration. Our data further demonstrated that brain tPA protein level determined by Western blot was significantly higher in tPA-treated sham rats and tPA-treated TBI rats 24 hr after intranasal administration than that in the saline-treated TBI rats (Fig. 1A and 1B, *p*<0.05). This result is consistent with the zymographic assay of tPA activity (Fig. 1C and 1D, *p*<0.05), showing a significantly higher tPA activity in the brain extracts 24 hr after tPA administration relative to the saline group. We measured plasmin activity of brain extracts with S-2251 (without addition of plasminogen in the assay system), demonstrating that plasmin activity was higher in tPA-treated animals than that in the saline-treated group at 2 days after TBI (Fig. 1E, *p*<0.05). Furthermore, the tPA activity measured by indirect amidolytic assay (Fig. 1F) is in line with that detected by direct zymography assay (Fig. 1C and 1D). Interestingly, the tPA protein level and activity in the injured brain were significantly higher than those in the sham brain although these animals received the same dose of tPA (Fig. 1 and 2, *p*<0.05).

### Subacute Intranasal tPA Administration Significantly Reduced Sensorimotor Deficits after TBI

The modified neurological severity score (mNSS) score was close to 12 in TBI rats (both the vehicle and tPA group) on Day 1 post controlled cortical impact (CCI)-induced TBI, indicating neurological functional deficits were comparable in all TBI rats (Fig. 2A). Spontaneous functional recovery occurred in the saline-treated animals. However, significantly improved functional recovery (that is, reduced mNSS score) was observed after TBI in the tPA group compared to the saline-treated group (*p*<0.05 on Days 14–35). tPA treatment also significantly reduced the frequency of right forelimb foot fault occurrence as compared to saline controls (Fig. 2B, *p*<0.05 on Days 14–35).

### Subacute Intranasal tPA Administration Significantly Improved Spatial Learning after TBI

Spatial learning was performed during the last five days (31–35 days post injury) prior to sacrifice using the modified Morris water maze (MWM) test, which is very sensitive to the hippocampal injury [24]. The greater the percentage of time the animals spend in the correct quadrant (i.e., Northeast, where the hidden platform was located) in the water maze, the better the spatial learning function. The percentage of time spent by sham rats in the correct quadrant increased significantly from 32–35 days after sham operation, as compared to time spent in the correct quadrant at Day 31 (Fig. 2C, *p*<0.05). The spatial learning function in the vehicle-treated TBI rats was significantly impaired compared to sham rats at 33–35 days after TBI (*p*<0.05). TBI rats treated with tPA showed significant improvement in spatial learning at 33–35 days when compared to the TBI rats treated with saline (*p*<0.05).

### Subacute Intranasal tPA Administration Significantly Increased Neuroblasts and Neurogenesis in the Dentate Gyrus after TBI

**Figure 3:**
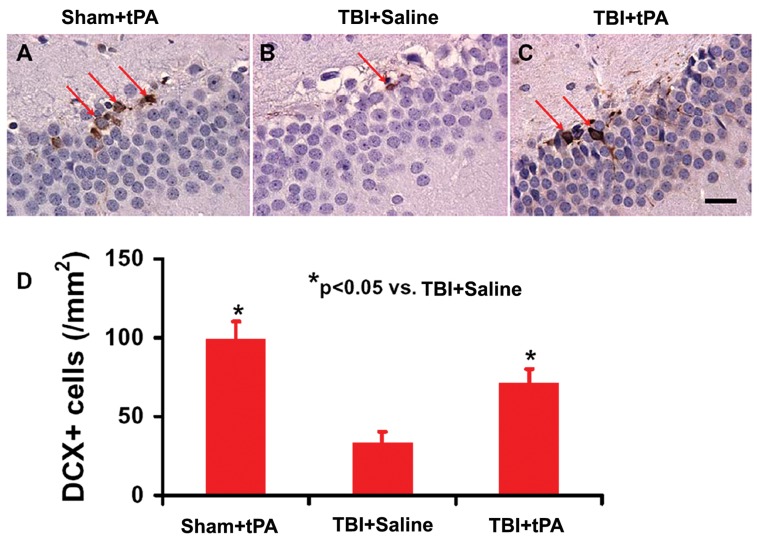
tPA effect on immature neurons after TBI. DCX staining (A–C). tPA significantly increased immature neurons identified with DCX-positive staining in the DG in rats examined 35 days after injury compared with the saline-treated group. The bar graph shows the number of DCX-positive cells. Scale bar  = 25 µm. *p<0.05 vs TBI+Saline. Data represent mean ± SD. n = 8 (rats/group).

**Figure 4:**
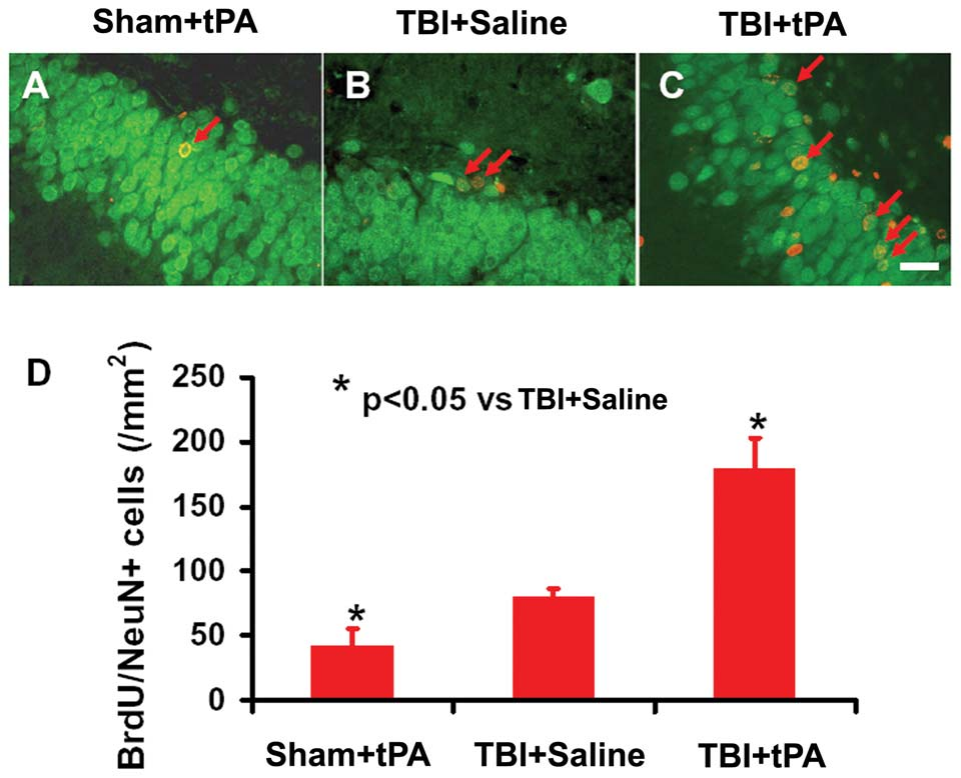
tPA effect on neurogenesis after TBI. Compared to the TBI+Saline group (B), tPA treatment (C) significantly increased newborn mature neurons identified with BrdU/NeuN double immunofluorescent staining in the DG 35 days post injury. The bar graph (D) shows the number of DCX-positive cells. Scale bar  = 25 µm. *p<0.05 vs TBI+Saline. Data represent mean ± SD. n = 8 (rats/ group).

**Figure 5:**
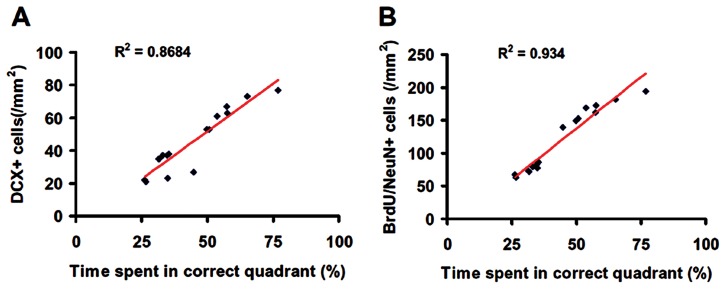
Correlation of the number of neuroblasts (A), and newborn neurons (B) with spatial learning. The line graph shows that spatial learning is significantly correlated with the number of DCX-positive cells (A) and to the number of newborn mature neurons (B) in the DG of the ipsilateral hippocampus in rats examined at 35 days after TBI and tPA treatment (*p*<0.05).

Immature newborn neurons in the DG were identified by doublecortin (DCX) staining. tPA significantly increased the number of DCX-positive cells compared to saline (Fig. 3, *p*<0.05). Double immunostaining for BrdU (proliferating marker) and NeuN (mature neuronal marker) was performed to identify newly generated neurons in the DG. tPA treatment significantly increased the number of newborn neurons in the DG compared to vehicle controls (Fig. 4, *p*<0.05). Our data further show that spatial learning was highly correlated to the number of DCX-positive cells (Fig. 5A, R ^2^ = 0.8684, *p*<0.05) and to the number of newborn mature neurons (Fig. 5B, R ^2^ = 0.934, *p*<0.05) in the DG of the ipsilateral hippocampus examined at Day 35 after TBI.

### Subacute Intranasal tPA Administration Significantly Promoted Midline-Crossing CST Axon Sprouting into the Denervated Side of the Cervical Spinal Cord after TBI

**Figure 6:**
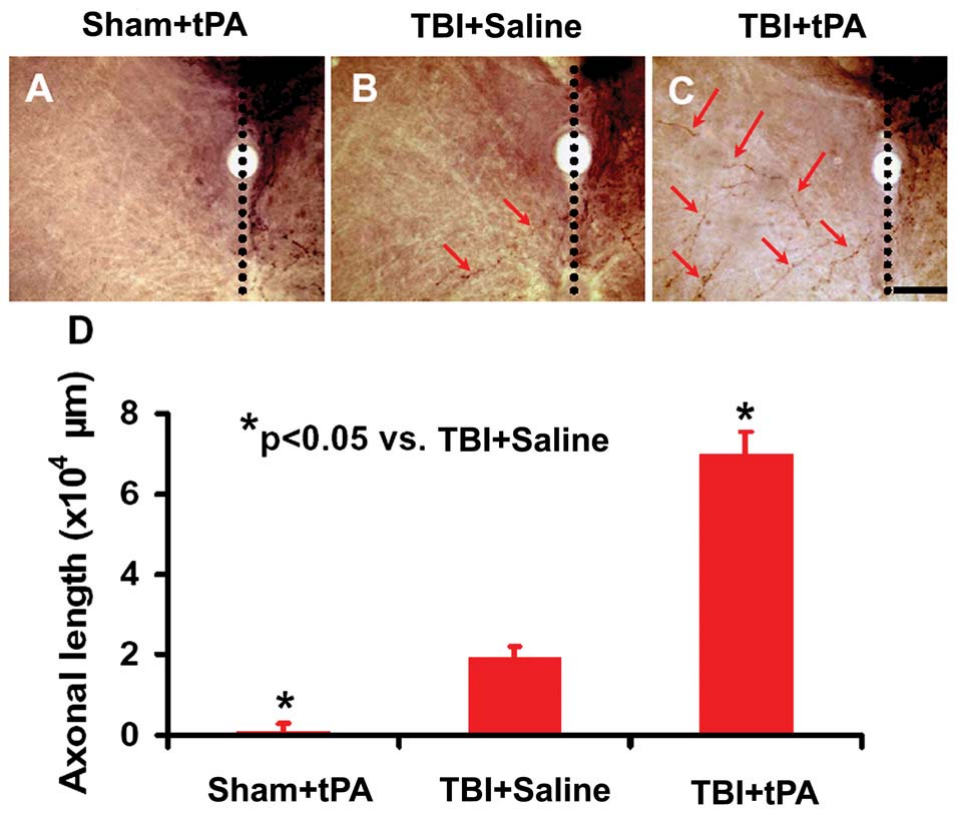
BDA-labeling of CST originating from the contralesional intact hemisphere. Representative images from the cervical spinal cord show BDA-labeled CST axons crossing the midline (arrows in B) and sprouting into the denervated side of the ventral gray matter in a rat after TBI. tPA treatment significantly increased the axon midline crossing (arrows in C). There were no obvious BDA-labeled axons observed in the opposite side of the cervical spinal cord in sham rats (A). Quantitative data (D) show that the number of contralesional CST in the denervated cervical gray matter was increased significantly by traumatic injury (*p*<0.05 vs. Sham+tPA) and tPA treatment (*p*<0.05 vs. TBI+Saline). Scale bar  = 50 µm. Data represent mean ± SD. n = 8 (rats/group).

**Figure 7:**
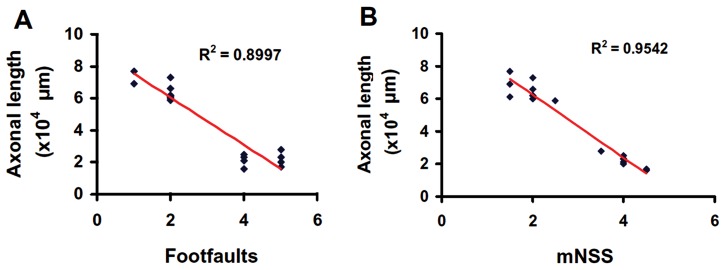
Correlation of the total length of axons crossing the midline at the cervical spinal cord with the right forelimb foot fault (A) and the mNSS score (B). The line graph shows that the total length of axonal crossing at the midline of the cervical level of the spinal cord is significantly reversely correlated with the incidence of forelimb footfault (A) and mNSS score (B) in rats examined at 35 days after TBI and tPA treatment (*p*<0.05).

To verify the neuroanatomical substrate of sensorimotor functional recovery after TBI, we injected an anterograde neuronal tracer biotinylated dextran amine (BDA) into the contralesional sensorimotor cortex to label the CST originating from this area. Our data showed that tPA treatment significantly increased CST axonal sprouting originating from the contralesional cortex crossing the midline to innervate the TBI-impaired cervical spinal cord (Fig. 6). Our data further show that the total length of axons crossing the midline at the cervical spinal cord was highly and inversely correlated to the incidence of forelimb footfaults examined at Day 35 after TBI (Fig. 7A, R ^2^ = 0.8997, *p*<0.05). Furthermore, the total length of axons crossing the midline at cervical spinal cord was highly and inversely correlated to the mNSS score assessed at Day 35 after TBI (Fig. 7B, R ^2^ = 0.9542, *p*<0.05).

### Electrophysiological Assessment of Functional Reorganization in the Contralesional Hemisphere after TBI

**Figure 8:**
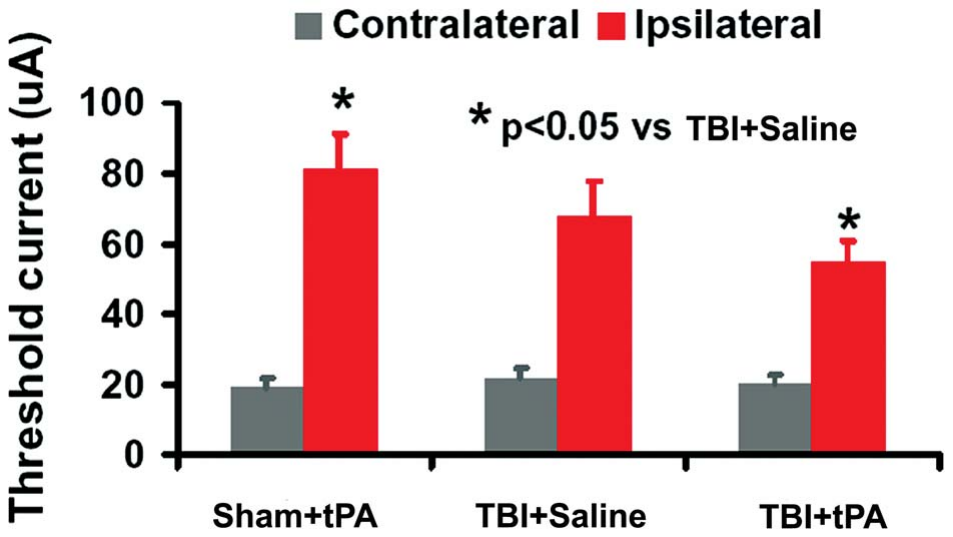
Mean threshold current levels needed to evoke forelimb movements on stimulation of the right cerebral motor cortex. For each animal, the threshold average was calculated from 4 stimulation points. ICMS of the motor cortex in normal adult rats evoked low threshold contralateral forelimb movements and high threshold ipsilateral movements. After TBI, the contralateral movement threshold (for left normal forelimb) was unchanged, whereas the ipsilateral movement threshold was decreased significantly at 5 weeks postinjury compared to sham (*p*<0.05). tPA treatment further reduced the threshold current needed for ipsilateral forelimb movement compared to the saline controls (*p*<0.05). Data represent mean ± SD. n = 8 (rats/group).

In this study, we performed ICMS on the right cortex and electromyogram (EMG) recording of the forelimb extensor muscles to test the contribution of the contralesional cortex to functional recovery in rats after TBI. The current threshold evoking movement in both forelimbs simultaneously was measured at 4 stimulation points. Current thresholds were at low levels to evoke left forelimb movement, and were comparable in all normal and TBI animals (Fig. 8, mean range 19–22 µA). In normal animals, threshold values evoking right forelimb movements were much higher than those required to evoke movement in the left forelimb (mean range 69–97 µA). However, 5 weeks after TBI the mean threshold in the contralesional right cortex eliciting movement in right-sided TBI-impaired forelimbs was significantly decreased (mean range 47–78 µA, *p*<0.05 vs Sham). tPA further reduced the mean threshold in the contralesional right cortex eliciting movement in the right-sided TBI-impaired forelimbs (mean range 45–63 µA, *p*<0.05 vs saline controls).

### Subacute Intranasal tPA Administration Reduced ProBDNF and Increased Mature BDNF Protein Level after TBI

**Figure 9:**
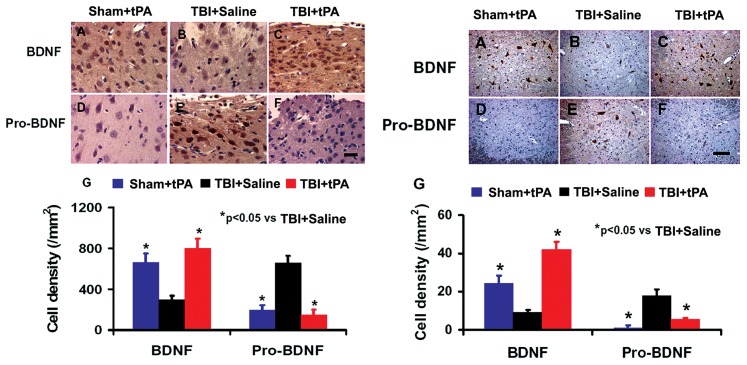
Immunostaining analysis of ProBDNF and mature BDNF-positive cells in the brain and spinal cord. Left panel: ipsilateral brain cortex; Right panel: denervated cervical spinal cord. *p<0.05 vs TBI+Saline. Scale bar  = 25 µm. Data represent mean ± SD. n = 4 (rats/group).

**Figure 10:**
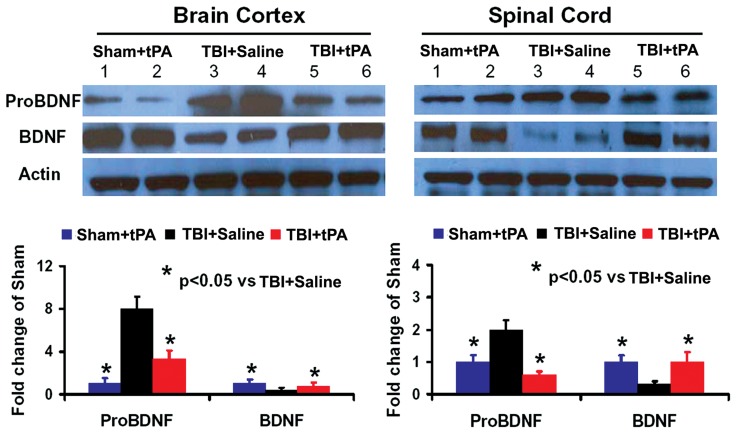
Western blot analysis of ProBDNF and mature BDNF protein levels in the brain and spinal cord. Left panel (ipsilateral brain cortex): Sham+tPA (1, 2), TBI+Saline (3, 4); 3: TBI+tPA (5, 6); Right panel (denervated cervical spinal cord): Sham+tPA (1, 2), TBI+Saline (3, 4), TBI+tPA (5, 6). *p<0.05 vs TBI+Saline. Data represent mean ± SD. n = 4 (rats/group).

To investigate the molecular mechanisms by which tPA promotes neuroplasticity and functional recovery, we examined the protein levels of proBDNF and BDNF in the cortex of injured brain and denervated cervical spinal cord in rats after TBI treated with tPA. TBI significantly increased ProBDNF protein level and decreased BDNF level in the cortex of injured brain and denervated cervical spinal cord while delayed intranasal tPA treatment significantly reduced the proBDNF protein level and increased BDNF level, as shown by immunostaining (Fig. 9) and Western blot analyses of pro/mature BDNF (Fig. 10), respectively. In the present study, although 4 rats were used for immunostaining, the statistical analysis using a one-way ANOVA followed by post hoc Student-Newman-Keuls tests reveals a significant difference in BDNF^+^ and ProBDNF^+^ cell density in brain cortex and spinal cord between the saline and tPA treatments. The number of animals used in this study is in agreement with previous studies in TBI using a minimal number of animals (n = 4 or less) for immunostaining by us [62] and many other investigators [63]–[67].

### Subacute Intranasal tPA Administration Did Not Alter Cortical Lesion Volume after TBI

**Figure 11:**
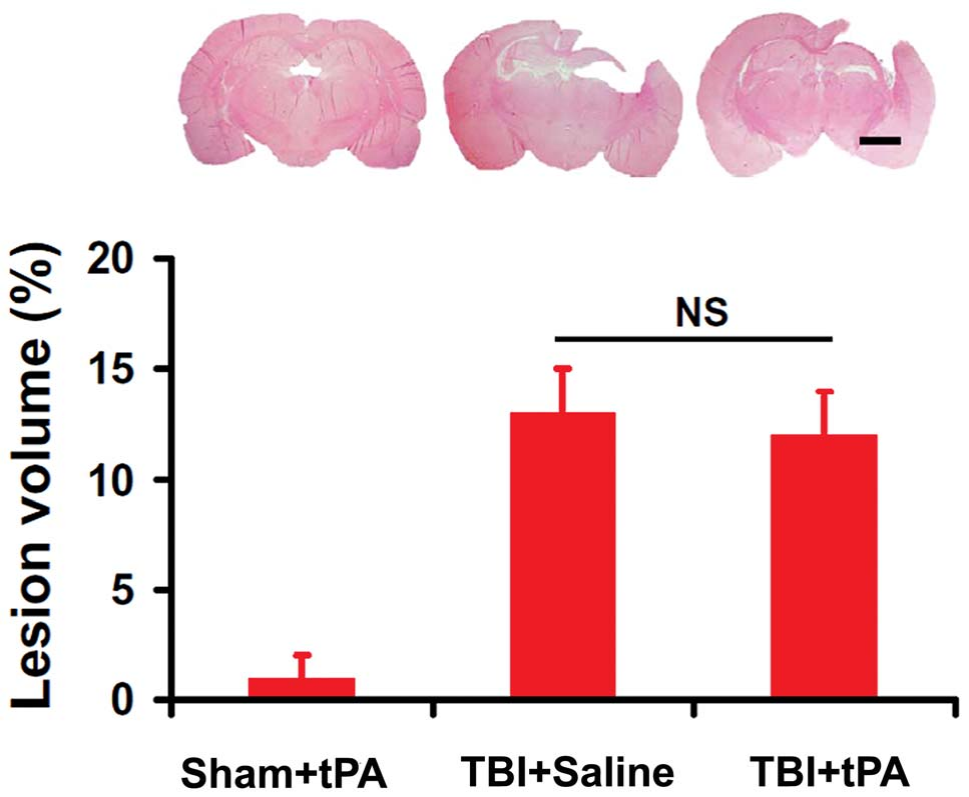
Cortical lesion volume after TBI and tPA treatment. The bar graph shows no significance (NS) in the cortical lesion volume between the TBI+Saline and TBI+tPA groups examined at 35 days post injury (*p*>0.05). Scale bar  = 2 mm. Data represent mean ± SD. n = 8 (rats/group).

Lesion volume was measured 35 days post TBI. Delayed intranasal tPA treatment initiated at 7 days post injury did not alter lesion volume compared to vehicle controls (Fig. 11, 12.8±2.4% for TBI+Saline rats vs 11.9±1.6% for TBI+tPA rats, *p*>0.05).

## Discussion

The present study demonstrates that subacute intranasal administration of tPA initiated 7 days after injury and repeated once at 14 days did not alter lesion size but significantly promoted neurological functional recovery in rats after TBI compared to treatment with saline. The subacute intranasal tPA treatment significantly increased immature and mature neurons in the DG of the ipsilateral hippocampus, which was significantly correlated with spatial learning detected by the MMW test. tPA also significantly enhanced axonal sprouting from the intact CST into the denervated side of the cervical spinal cord, which was significantly correlated with sensorimotor functional recovery assessed by the footfault and mNSS tests. ICMS on the right cortex and EMG recording from the forelimb extensor muscles imply that new neuronal connections were established between the intact cerebral hemisphere and the impaired forelimb after TBI. Our findings further indicate that tPA reduced proBDNF and increased BDNF protein level in the injured brain and denervated cervical spinal cord. Our data demonstrated that levels of tPA protein and activity in the brain extracts 24 hr after intranasal administration of tPA in the tPA-treated animals were significantly higher than those in the saline-treated group. Taken together, our data suggest that subacute intranasal tPA treatment improves functional recovery, at least in part, by enhancing endogenous brain neurogenesis and CST axonal sprouting, which is associated with tPA/plasmin-dependent maturation of BDNF.

Neuroprotective approaches have historically been dominated by targeting neuron-based injury mechanisms as the primary or even exclusive focus of the therapeutic strategy [68]. The failure of all recent clinical trials for TBI targeting neuroprotection suggests that we need to consider new approaches to the study and development of therapeutic agents. The aim of this study was to test whether intranasal tPA administration would be a new noninvasive neurorestorative treatment for subacute TBI. A wide distribution of tPA biosynthesis in the brain is associated with different actions of tPA, such as facilitating synaptic plasticity [69] and axonal regeneration [70], which may contribute to neural repair after injury. After acute ischemic stroke, the thrombolytic effect of intravenous tPA administration in the intravascular space is beneficial, whereas its extravascular effect on ischemic neurons may be deleterious. It was documented that direct intracerebral perfusion of the tPA solution (15 and 30 µmol/L) via a microdialysis probe caused brain tissue damage in normal rats [71]. Interestingly, acute (10 min post injury) intracortical injection of the t-PA: plasminogen activator inhibitor 1 complex promotes neurovascular injury in mouse models of TBI by upregulating matrix metalloproteinase-3 [72]. The tPA dose, administration time, and route used in our present study was chosen based on our previous study in stroke rats [20], which shows robust therapeutic effects in rats after stroke. In the present study, we focused on the efficacy of delayed (7 days post TBI) intranasal administration of tPA in an animal model of TBI. Reduction in lesion volume after a cortical controlled impact injury is often used to imply recovery of function in animal models of TBI after neuroprotective treatment [1]. The finding from the present study shows that delayed intranasal tPA therapy without reduction of the lesion is capable of improving functional recovery. However, high concentrations of tPA via direct intracerebral perfusion of the tPA solution cause brain tissue damage in normal rats [71]. Here, we take advantage of intranasal tPA delivery during the subacute phase after TBI. We demonstrated that intranasal administration of h-r-tPA increases brain tPA protein level and tPA activity as measured by Western blot, ELISA, amidolytic assay, and zymography although the exact amount of active h-r-tPA entering the brain via the intranasal route is actually unknown. We do not expect that a vast amount of active tPA was delivered intranasally to the brain after TBI. It is possible that only small amounts of active tPA are needed to produce the beneficial effects as reported in this study. Interestingly, the tPA protein level and activity in the injured brain (TBI+tPA group) were significantly higher than those in the Sham+tPA rat brain although these animals received the same dose of tPA. The reason for this difference remains unclear. Although we did not determine the amount of tPA administered which actually entered rat brain, the increase in brain tPA level was not attributed to endogenous tPA after TBI because the tPA level in the injured rat without tPA treatment (TBI+Saline group) was low. We cannot exclude the possibilities that the difference in brain tPA activity may result from the more tPA delivery to the injured brain than to the normal brain or likely reduced levels of specific tPA inhibitors including plasminogen activator inhibitor-1 (PAI-1) and neuroserpin [73], investigation of which is warranted. Our data provide a proof-of-principle supporting the hypothesis that intranasal tPA administration in rats during the subacute phase after TBI provides neurorestorative effects by promoting neuroplasticity (neurogenesis and axonal sprouting) and enhancing neurological recovery, while not reducing the lesion volume.

The role of tPA after brain injury is not fully understood. It is known that tPA plays a detrimental role in acute TBI [18], [72], [74]–[76]. For example, a previous study demonstrated that acute intravenous injection of free tPA into rats 15 min after TBI caused significantly larger cortical injuries and greater cerebral hemorrhage [18]. However, 30 min after TBI, intravenous injection of a mutant tPA protein tPA-S481A lacking the catalytic activity but maintaining other functions of the wild type tPA reduced neurotoxicity caused by untoward NMDA-receptor activation mediated by increased tPA and glutamate [74], [75], [77]. Further investigation of the beneficial effects of mutant tPA on functional recovery is warranted in animal models of TBI. Our present study differs from acute studies in that we deliver tPA initially at 7 days post TBI, intranasally. Although we did not directly measure cerebral hemorrhage, our delayed intranasal tPA administration did not alter lesion size but promoted neuroplasticity including increased brain neurogenesis and CST axonal sprouting. In our previous stroke study, we have shown that intranasal tPA administration in the subacute phase starting 7 days after ischemic stroke in rats and repeated 14 days significantly enhances sensorimotor functional recovery by promoting axonal sprouting into the denervated side of the spinal cord [20]. No adverse effects or hemorrhage were observed in the intranasally delivered tPA-treated stroke animals. In addition, increased endogenous tPA has been implicated to promote brain repair and functional recovery with neurorestorative therapies in rats after subacute brain injuries including stroke [11], [78] and TBI [12]. These results indicate that tPA is beneficial if given intranasally at the subacute stage after TBI. In the present study, tPA was administered at 7 and 14 days postinjury (DPI); however, the critical period for functional recovery (Fig. 1A, 1B) was clearly between 7 and 14 DPI. After that period, the slopes of the functional recovery of TBI +Saline and TBI +tPA rats are similar, suggesting that administration at 14 DPI may not be necessary and that the first administration at 7 DPI is sufficient to significantly enhance neurological recovery.

Our ICMS-EMG data indicate that new neuronal connections are established between the intact cerebral hemisphere and the impaired side of the body after TBI and these neuronal connections are further enhanced by tPA treatment. ICMS has been used to define movement representations in motor cortex [79] and to evaluate changes in plasticity in the motor cortex following motor system lesion [80] by applying stimulative current on cortical pyramidal neurons through efferent connections to the spinal motoneurons to elicit limb movement. In the present study, we performed ICMS on the right contralesional cortex and EMG recording of the bilateral forelimb extensor muscles. The lower threshold current evoking ipsilateral forelimb movement suggested an increased neuronal coupling between the intact motor cortex and the spinal motor neurons in the denervated side of the ventral horn. Our ICMS-EMG data are consistent with increased BDA-labeled CST axonal sprouting originating from the contralesional hemisphere at the cervical level. In the present study, using BDA labeling and ICMS-EMG recording, we have found that intranasal tPA facilitates the structural and functional links between the denervated cervical spinal cord and intact motor cortex after subacute TBI. However, other descending pathways in the spinal cord such as the corticorubral tract may also undergo remodeling, which may be beneficial to functional recovery. We cannot exclude this possibility.

Continuous generation of new neurons from neural stem/progenitor cells in the subgranular zone of the DG is essential for hippocampus-dependent learning and memory [81]. Increased neurogenesis occurs in the hippocampus of adult animals and humans after TBI, which may contribute to spontaneous hippocampus-dependent functional recovery [6], [82], [83]. Preclinical data from us and others have shown that a large proportion of newly generated cells in the DG die within one month in rodents after TBI [82], [84]. In the present study, compared to saline controls, tPA treatment increased the number of immature neurons (DCX^+^ cells) and mature neurons (BrdU/NeuN^+^ cells) in the DG examined at 35 days after TBI, suggesting that tPA treatment stimulates and supports adult neurogenesis in the hippocampus following TBI. Previous studies show that adult newborn neurons in the DG display typical features of mature granule cells within 4 weeks of their birth [85], [86]. At 1 month, these cells have the morphological and physiological characteristics of granule cells, and their full maturation and incorporation into functional circuits appears to be a prolonged process [87]. Indeed, newborn neuron physiology, plasticity, and circuitry may continually evolve for at least 3 months [87]. Thus, further investigation of the long-term effects of tPA treatment is warranted. Taken together, these results suggest that tPA has therapeutic potential as a noninvasive neurorestorative agent to improve functional recovery during the subacute phase after brain injury.

The molecular mechanisms underlying tPA-mediated brain and spinal cord neuroplasticity associated with functional recovery after subacute TBI remain unclear. Our present study demonstrates that tPA reduces proBDNF level and increases mature BDNF level in the injured brain and denervated cervical spinal cord, indicating a potential conversion of proBDNF to BDNF after tPA treatment. Our previous study indicates that intranasal delivery is an efficacious method to deliver tPA into the rodent brain [20]. tPA is able to activate plasminogen to plasmin, which converts the precursor proBDNF to the mature BDNF, and this conversion is critical for neuroplasticity and neuronal function [14], [16], [17], [58], [88], [89]. Acute application of mature BDNF facilitates long-term potentiation (LTP) in the hippocampus [90], while inhibition of BDNF activity (by gene knockout or functional blocking using BDNF antibody) attenuates hippocampal LTP [90], [91]. BDNF is involved in regulating the survival of adult-born immature neurons in the hippocampus following TBI [92]. Conversely, proneurotrophins often have biological effects that oppose those of mature neurotrophins [93], [94]. For example, proBDNF has an opposing role in neurite outgrowth to that of mature BDNF, proBDNF collapses neurite outgrowth of primary neurons [14] and negatively regulates neuronal remodeling and synaptic plasticity in the hippocampus [95]. Increasing evidence indicates that dysregulation of BDNF occurs after TBI, and induction of BDNF and activation of its intracellular receptors promotes neural regeneration, reconnection, and dendritic sprouting, and improves synaptic efficacy [96]. BDNF is unable to cross the intact blood brain barrier in vivo, and it is not effective when given intravenously [97]. Treatment approaches including exercises that enhance endogenous BDNF have the potential to restore neural connectivity and functional recovery [96], [98]. In addition, chondroitin sulfate proteoglycans (CSPGs) play a pivotal role in many neuronal growth mechanisms following injury to the spinal cord or brain [99]. tPA/plasmin degrades CSPGs including neurocan and phosphacan in the brain and promotes neurite reorganization after seizures [100]. tPA knockout mice exhibit attenuated neurite outgrowth and blunted sensory and motor recovery after spinal cord injury despite chondroitinase ABC (ChABC) treatment, which degrades the sugar chains of CSPGs and allows for synaptic plasticity [101]. The tPA/plasmin system specifically degrades NG2 and phosphacan after ChABC cleavage in vivo [101]. These findings show that the tPA/plasmin cascade may act downstream of ChABC to allow for synaptic plasticity improvement which enhances functional recovery after neural injury. We cannot exclude the effect of tPA/plasmin on CSPG degradation [101]. Additional studies of tPA/plasmin/CSPG interactions are warranted in TBI after tPA treatment.

Plasmin represents the critical enzyme that drives axonal plasticity and regeneration via BDNF effects and/or by degrading CSPGs [101]. Our current findings demonstrate that tPA enters the brain after intranasal administration and remains active 24 hr after the treatment, which indicates that the tPA/plasmin system is an important pathway responsible for maturation of BDNF likely by converting pro-BDNF to BDNF, suggested by our Western blot and immunostaining data. However, we did not investigate other possible pathways for BDNF maturation in the present study, and cannot exclude the tPA/plasmin-independent pathway. tPA is capable of regulating brain BDNF synthesis through a plasmin-independent effect mediating by N-methyl-D-aspartate (NMDA) receptor signaling [102]. For example, intravenous administration of recombinant tPA (10 mg/kg) induced an increase in hippocampal recombinant tPA and mature BDNF expression in normal adult male Wistar rats 2 hr and 24 hr after tPA administration but did not increase hippocampal plasmin activity; while MK801 an NMDA receptor antagonist completely abolished the rise in mature BDNF expression induced by tPA [102]. Their data strongly suggest that exogenous tPA increases mature BDNF expression in the hippocampus through NMDA receptor activation, which is independent of plasmin activity. In contrast, our data suggest that intranasal administration of tPA increased plasmin activity in the brain measured by amidolytic assay. Intranasal administration of tPA targeting the tPA/BDNF system opens a new avenue for subacute treatment of TBI. In addition, plasmin formation can also be generated by urokinase-type plasminogen activator (uPA) [103]. It is warranted to compare the effects of intranasal treatment with uPA with those of tPA after subacute TBI.

## Conclusion

The present study demonstrates that intranasal administration of tPA at the subacute phase after TBI significantly improves sensorimotor and cognitive functional recovery and enhances brain neurogenesis and CST compensatory axonal remodeling, which is likely associated with tPA/plasmin-dependent maturation of BDNF. These findings suggest that tPA holds potential for a noninvasive neurorestorative treatment for subacute TBI. However, further studies are warranted to discover the optimal dose and therapeutic window of intranasal tPA administration after TBI, and to investigate the molecular mechanisms underlying the therapeutic effects as well as potential side effects including brain hemorrhage.
